# Activation of Peracetic Acid with Lanthanum Cobaltite Perovskite for Sulfamethoxazole Degradation under a Neutral pH: The Contribution of Organic Radicals

**DOI:** 10.3390/molecules25122725

**Published:** 2020-06-12

**Authors:** Xuefei Zhou, Haowei Wu, Longlong Zhang, Bowen Liang, Xiaoqi Sun, Jiabin Chen

**Affiliations:** State Key Laboratory of Pollution Control and Resources Reuse, College of Environmental Science and Engineering, Tongji University, Shanghai 200092, China; zhouxuefei@tongji.edu.cn (X.Z.); wuhaowei@tongji.edu.cn (H.W.); 1810332@tongji.edu.cn (L.Z.); 1750564@tongji.edu.cn (B.L.); 1754021@tongji.edu.cn (X.S.)

**Keywords:** advanced oxidation processes (AOPs), peracetic acid, LaCoO_3_, organic radicals

## Abstract

Advanced oxidation processes (AOPs) are effective ways to degrade refractory organic contaminants, relying on the generation of inorganic radicals (e.g., ^•^OH and SO_4_^•−^). Herein, a novel AOP with organic radicals (R-O^•^) was reported to degrade contaminants. Lanthanum cobaltite perovskite (LaCoO_3_) was used to activate peracetic acid (PAA) for organic radical generation to degrade sulfamethoxazole (SMX). The results show that LaCoO_3_ exhibited an excellent performance on PAA activation and SMX degradation at neutral pH, with low cobalt leaching. Meanwhile, LaCoO_3_ also showed an excellent reusability during PAA activation. In-depth investigation confirmed CH_3_C(O)O^•^ and CH_3_C(O)OO^•^ as the key reactive species for SMX degradation in LaCoO_3_/PAA system. The presence of Cl^−^ (1–100 mM) slightly inhibited the degradation of SMX in the LaCoO_3_/PAA system, whereas the addition of HCO_3_^−^ (0.1–1 mM) and humic aid (1–10 mg/L) could significantly inhibit SMX degradation. This work highlights the generation of organic radicals via the heterogeneous activation of PAA and thus provides a promising way to destruct contaminants in wastewater treatment.

## 1. Introduction

Advanced oxidation processes (AOPs) have been extensively used to degrade toxic and bio-recalcitrant organic contaminants in wastewater treatment [1,2,3,4,5,6,7,8]. The commonly used AOPs are generally based on the in situ generation of highly reactive inorganic radicals, such as the hydroxyl radical (HO^•^) and the sulfate radical (SO_4_^•−^). They are usually generated through the homogeneous cleavage of the peroxide bond in inorganic peroxides, such as hydrogen peroxide (H_2_O_2_), peroxydisulfate (PDS) and peroxymonosulfate (PMS) [9]. The inorganic radicals possess high oxidation potential (E^0^ = 2.5–3.1 V for SO_4_^•-^ and E^0^ = 1.9–2.7 V for HO^•^) [10] and thus can effectively destruct a wide range of organic contaminants with the second-order rate constants ranging from 10^5^ to 10^9^ M^−1^ s^−1^ for SO_4_^•−^ [11] and from 10^7^ to 10^10^ M^−1^ s^−1^ for HO^•^ [12]. However, the short half-life (30–40 μs for SO_4_^•−^, 1 μs for HO^•^) of inorganic radicals may reduce the possibility of their interaction with the target contaminants [13,14]. On the other hand, the non-selectivity of inorganic radicals render them susceptible to consumption by the water matrices, e.g., anions and natural organic matters. These drawbacks reduce the degradation efficiency of target contaminants by inorganic radicals in real wastewater treatment [15].

Recently, an emerging AOP mainly based on the organic radicals (R-O^•^) has become an attractive alternative for organic contaminant degradation in wastewater treatment. Organic radicals are commonly generated from the activation of peroxide bond in the organic peroxy acid, such as peracetic acid (PAA, CH_3_C(O)OOH) [16,17]. PAA is known as a broad-spectrum antimicrobial agent and has been widely used as an alternative to conventional chlorine disinfectants in wastewater disinfection, owing to the advantages of PAA, such as strong oxidation power (redox potential ranging from 1.06 to 1.96 V) [18] and a low potential of harmful disinfection byproduct generation [19,20,21]. PAA is produced by the reaction of acetic acid and H_2_O_2_ in the presence of a strong acid catalyst, such as sulfuric acid [22,23,24], and thus the commercial PAA solution is an equilibrium solution with acetic acid and H_2_O_2_ according to Equation (1).

(1) $${\mathrm{CH}}_{3}{C(=O)\mathrm{OOH}+H}_{2}O↔{\mathrm{CH}}_{3}{C(=O)\mathrm{OH}+H}_{2}O_{2}$$

PAA can directly oxidize some organic contaminants in wastewater treatment, e.g., amino acid [25] and β-lactam antibiotics [26]. After the introduction of intensive energy (e.g., heat and UV) or catalysts, the peroxide bond in PAA can be activated to produce reactive species (e.g., HO^•^ and R-O^•^) [19,27,28,29]. Compared with the inorganic peroxides, e.g., H_2_O_2_ (213 kJ mol^−1^), PAA has a weaker -O–O- bond (159 kJ mol^−1^ for PAA [26,30]), which can be easily activated for intensive radical generation to attack target contaminants, such as pharmaceuticals [19,31], phenols [27,32], and dyes [33]. Therefore, PAA shows a great potential to be an ideal alternative to H_2_O_2_ in AOPs and the development of efficient and environmentally friendly methods to activate PAA is of great significance.

Many attempts have been made to develop effective strategies for the activation of PAA. Among them, transition metal ions have been reported as the most viable activators. For example, iron and cobalt ions showed a great performance for the activation of PAA to degrade organic contaminants [31,34]. However, one of the serious hindrances to this homogeneous processes was the poor reusability and potential toxicity of some metal ions [35]. A heterogeneous catalyst containing cobalt with low cobalt leaching and high reusability is expected to overcome these problems. In this work, a Co-based perovskite (LaCoO_3_) was used to activate PAA to eliminate recalcitrant contaminants. Perovskite, with the ABO_3_ structure, has recently attracted considerable interests in the field of material science and heterogeneous catalysis [36,37], due to the flexible chemical composition, element abundance and structural stability. In particular, perovskite oxides are regarded as excellent catalysts in wastewater treatment. For example, LaCoO_3_ has been used as a catalyst to activate PMS or H_2_O_2_ for contaminant degradation [36,38,39,40]. However, the performance of LaCoO_3_ on PAA activation and the mechanisms underlying the LaCoO_3_^−^activated PAA reaction are still unknown.

Herein, LaCoO_3_ perovskite was synthesized via the sol-gel method and then used to activate PAA to degrade sulfamethoxazole (SMX). The aims of this study were: 1) to investigate the performance of LaCoO_3_/PAA on the degradation of SMX; 2) to evaluate the impact of water matrices on SMX degradation in the activated PAA process; 3) to identify the reactive radical species dominated in the contaminant degradation; 4) to reveal the activation mechanism of PAA by LaCoO_3_. To our best knowledge, this study is among the first to investigate the activation of PAA by perovskite and elucidate the mechanism for organic radical generation.

## 2. Results

### 2.1. Characterization of LaCoO_3_

**Figure 1:**
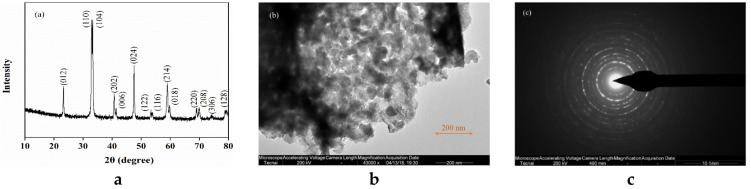
(**a**) X-ray powder diffraction (XRD) pattern, (**b**) transmission electron microscope (TEM) image and (**c**) selected-area electron diffraction (SAED) image of the LaCoO_3_.

Figure 1a shows the X-ray powder diffraction (XRD) pattern of LaCoO_3_. The diffraction peaks at 2θ values of 23.30°, 32.83°, 33.29°, 39.76°, 40.13°, 47.68°, 52.71°, 53.66°, 59.08°, 59.93° and 68.95° correspond to miller indices values of (012), (110), (104), (202), (006), (024), (122), (116), (214), (018) and (220), respectively [38]. In addition, the morphological properties of LaCoO_3_ was analyzed by Transmission Electron Microscope (TEM). The TEM image (Figure 1b) shows a disordered microstructure of the porous LaCoO_3_ nanospheres, with the average particle size approximately 23 nm according to the transmission electron microscope (TEM) image. The selected-area electron diffraction (SAED) pattern in Figure 1c revealed that the LaCoO_3_ nanospheres were polycrystalline. The Brunauer–Emmett–Teller (BET) area of the sample was obtained to be 14 m^2^/g and the zeta potential of LaCoO_3_ was determined to be 6.1.

### 2.2. Degradation of SMX by PAA Activated with LaCoO_3_

**Figure 2:**
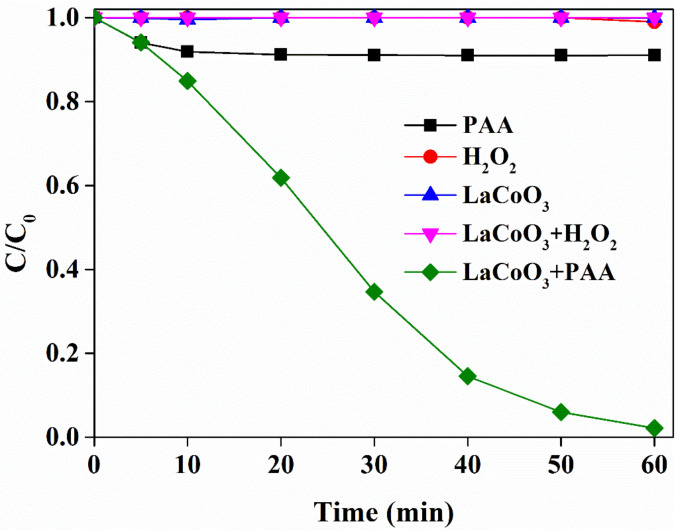
Sulfamethoxazole (SMX) degradation in different systems. LaCoO_3_ = 20 mg/L, SMX = 50 μM, peracetic acid (PAA) (or H_2_O_2_) = 660 μM, pH 7, 25 °C.

The prepared LaCoO_3_ was used to activate PAA for the degradation of SMX. As shown in Figure 2, the loss of SMX was not observed in the presence of LaCoO_3_ alone, indicating the negligible adsorption of SMX on LaCoO_3_. SMX was slightly degraded after the addition of PAA alone, suggesting the slight reactivity of PAA towards SMX. However, SMX could be almost completely degraded within 60 min in the presence of PAA and LaCoO_3_; hence, LaCoO_3_ and PAA showed a synergistic effect on SMX degradation. It should be addressed that the removal efficiency of SMX achieved by LaCoO_3_/PAA in this study was comparable or even higher than many published studies [41,42,43,44,45,46], and with much less catalyst addition. For example, Yan et al. [46] prepared CuO@Al_2_O_3_ to activate PMS and approximately 90% of SMX was removed under similar conditions ([SMX]_0_ = 39.5 μM, [PMS_]0_ = 0.4 mM, pH 6.2) but a large catalyst dosage ([cata]_0_ = 500 mg/L). Lalas et al. [45] reported that complete SMX degradation was obtained within 90 min through persulfate with 2 g immobilized CuOx catalyst. The LaCoO_3_ was reported as an excellent catalyst for the activation of peroxides, such as H_2_O_2_ and PMS, to generate reactive species to degrade contaminants [39,47,48]. It was most likely to activate PAA to degrade SMX. It is noted that commercial PAA solution always contains a certain amount of H_2_O_2_ during preparation; we thus evaluated the contribution of H_2_O_2_ to SMX degradation in the LaCoO3/PAA system. As Figure 2 shows, the degradation of SMX was negligible in the presence of H_2_O_2_, or H_2_O_2_/LaCoO_3_, where H_2_O_2_ concentration was equivalent to that in PAA solution. Hence, the degradation of SMX in LaCoO_3_/PAA was not affected by H_2_O_2_ in PAA solution. Meanwhile, this result also indicated that PAA was more easily activated by LaCoO_3_ than H_2_O_2_. Indeed, the peroxide bond energy in PAA was reported to be 159 kJ mol^−1^, which is lower than that in H_2_O_2_ (213 kJ mol^−1^) [31]. Hence, the dissociation of peroxide bond in PAA to generate reactive species is thermally feasible and even likely more easily than H_2_O_2_.

### 2.3. Effects of Catalyst Loadings

**Figure 3:**
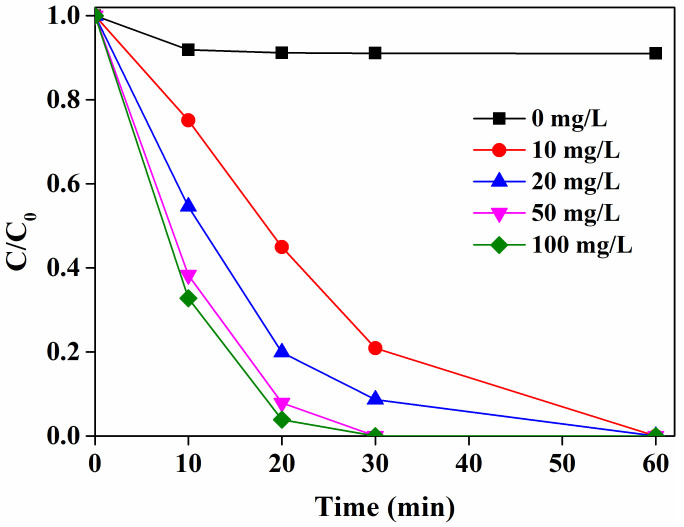
Effect of LaCoO_3_ loading on SMX degradation in the LaCoO_3_/PAA system. SMX = 50 μM, PAA = 660 μM, pH 7, 25 °C.

The effect of catalyst loading on the SMX degradation in the LaCoO_3_/PAA system was evaluated and the results are shown in Figure 3. Noting that the adsorption of SMX on LaCoO_3_ was negligible even in the presence of 0.1g/L LaCoO_3_ (data not shown), the removal of SMX could be attributed to the oxidative degradation rather than adsorption in the LaCoO_3_/PAA system. The degradation of SMX was gradually increased with the increasing loadings of LaCoO_3_ from 10 to 50 mg/L. A higher loading of LaCoO_3_ could supply more active sites for PAA to generate reactive species for contaminant degradation. When the LaCoO_3_ loading further increased to 100 mg/L, the degradation of SMX was only slightly increased. Hence, high loading of LaCoO_3_ had excess reactive sites for PAA activation, and even likely to quench the reactive species generated from PAA activation. Therefore, the excess loading of LaCoO_3_ could not further enhance the PAA activation.

### 2.4. Activation Mechanism

**Figure 4:**
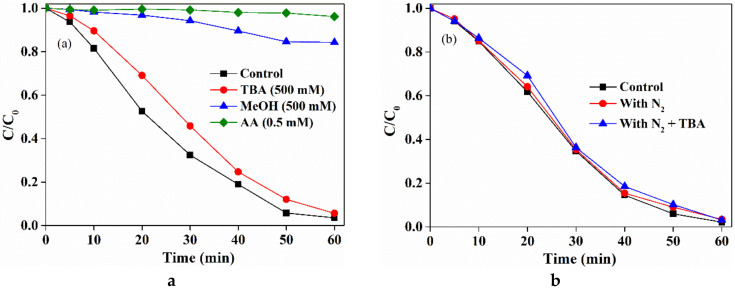
(**a**) Effect of ascorbic acid (AA), *tert*-butanol (TBA) and MeOH, and (**b**) Dissolved oxygen (DO) on the degradation of SMX in the LaCoO_3_/PAA system. LaCoO_3_ = 20 mg/L, SMX = 50 μM, PAA = 660 μM, pH 7, 25 °C.

To get insight into the degradation of SMX in the LaCoO_3_/PAA system, the reactive species generated from PAA activation by LaCoO_3_ was comprehensively studied. Ascorbic acid (AA) is a common scavenger for reactive radicals, and thus is widely used for investigating radical contributions in contaminant degradation [33]. As can be seen in Figure 4a, the presence of AA significantly inhibited the degradation of SMX in the LaCoO_3_/PAA system, and the degradation rates decreased from 98.9% to 3.8% with the concentration of AA increased from 0 to 0.5 mM. This result indicated that the radical species contributed to the degradatoin of SMX in the LaCoO_3_/PAA system.

Generally, the activation of PAA could generate various reactive radical species, mainly including ^•^OH, CH_3_C(=O)O^•^, CH_3_C(=O)OO^•^, CH_3_^•^ and CH_3_O_2_^•^ (Equations (2–6)) [30].

(2) $${\mathrm{CH}}_{3}C(=O)\mathrm{OOH}\;\Leftrightarrow {\;\mathrm{CH}}_{3}{C(=O)O}^{•}{+}^{•}\mathrm{OH}$$

(3) $${\mathrm{CH}}_{3}{C(=O)O}^{•}\;\to {\;\mathrm{CH}}_{3}{}^{•}{+\mathrm{CO}}_{2}$$

(4) $${\mathrm{CH}}_{3}{}^{•}{+O}_{2}\;\to {\;\mathrm{CH}}_{3}O_{2}{}^{•}$$

(5) $${\mathrm{CH}}_{3}{C(=O)\mathrm{OOH}+}^{•}\mathrm{OH}\;\to {\;\mathrm{CH}}_{3}{C(=O)\mathrm{OO}}^{•}{+H}_{2}O$$

(6) $${\mathrm{CH}}_{3}{C(=O)\mathrm{OOH}+\mathrm{CH}}_{3}{C(=O)O}^{•}\;\to {\;\mathrm{CH}}_{3}{C(=O)\mathrm{OO}}^{•}{+\mathrm{CH}}_{3}\mathrm{COOH}$$

Some radical species might contribute to the contaminant degradation in the activated PAA system. For example, ^•^OH contributed to the degradation of carbamazepine and ibuprofen in the UV/PAA system, whereas a strong reactivity of CH_3_C(=O)O^•^ and/or CH_3_C(=O)OO^•^ was observed in the oxidation of certain naphthyl compounds, such as naproxen and 2-naphthoxyacetic acid [19].

The quenching experiments were further conducted to reveal which radicals were the main reactive species contributed to SMX degradation in the LaCoO_3_/PAA system. *Tert*-butanol (TBA) is a desired quenching agent to distinguish the contribution of ^•^OH in the activation of PAA because TBA is highly reactive towards ^•^OH (k = 6.0 × 10^8^ M^−1^ s^−1^), but was inert towards other R-O^•^. Noting that the rate constants between SMX and ^•^OH is 7.9 × 10^9^ M^−1^ s^−1^, the contribution of ^•^OH can be almost completely consumed when the concentration of TBA is 1000 times higher than that of SMX. As shown in Figure 4a, the presence of excess TBA (500 mM) slightly inhibited the degradation of SMX in PAA/ LaCoO_3_ system, indicating that ^•^OH played a negligible role in LaCoO_3_/PAA system for SMX degradation. Moreover, MeOH is regarded as a common scavenger for ^•^OH (k = 6.0 × 10^8^ M^−1^s^−1^), and has been recently reported as a scavenger for organic radicals (R-O^•^) in the activated PAA [31]. Therefore, the addition of excess MeOH could be used to evaluate the contributions of R-O^•^ under the premise of TBA quenching results. In contrast to TBA, the addition of MeOH could significantly suppress the degradation of SMX in the LaCoO_3_/PAA system, indicating that the formed R-O^•^ might play a major role in SMX degradation.

To further validate the contributions of CH_3_^•^ and CH_3_O_2_^•^, N_2_ was pumped into the reaction solution. Because CH_3_^•^ can quickly react with oxygen to form CH_3_O_2_^•^ (Equation (4), k = (2.8–4.1) × 10^9^ M^−1^ s^−1^), the contribution of CH_3_^•^ and CH_3_O_2_^•^ could be determined when the dissolved oxygen (DO) was excluded in the presence of high purity N_2_. The concentration of DO was determined to decrease from 4.95 mg/L to 0.21 mg/L after N_2_ purging. As shown in Figure 4b, the purging of N_2_ showed a negligible inhibition effect on SMX degradation, suggesting that the contributions of CH_3_^•^ and CH_3_O_2_^•^ to SMX degradation could be ignored. Therefore, CH_3_C(=O)O^•^ and CH_3_C(=O)OO^•^ were speculated to be the primary organic radical species that contributed to SMX degradation in the LaCoO_3_/PAA system.

**Figure 5:**
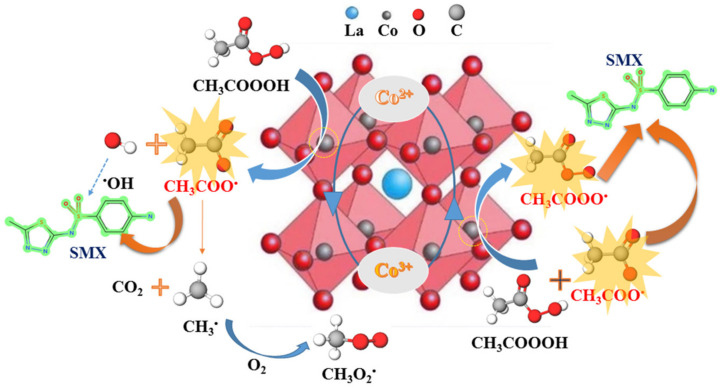
Proposed mechanism of PAA activated by LaCoO_3_.

The leaching of Co from LaCoO_3_ during the reaction was detected. After 60 min, the leached Co in the solution was determined to be 0.13 mg/L, which is much lower than the required concentration (< 1 mg/L) in the Environmental Quality Standards for Surface Water in China. In order to further investigate whether the leached Co contributed to the PAA activation for SMX degradation, PAA was introduced to the leached solution, and the result show that the degradation of SMX was not observed. Hence, the degradation of SMX in LaCoO_3_/PAA originated from the heterogeneous activation of PAA by LaCoO_3_ rather than the homogeneous activation with the leached Co ions. The heterogeneous activation of PAA with LaCoO_3_ was proposed in Figure 5. PAA was first adsorbed on the surface of LaCoO_3_, and then activated by the Co on the B site of perovskite to generate organic radicals, e.g., CH_3_COO^•^, which could act as an oxidant for SMX degradation. Meanwhile, Co(II) was oxidized to Co(III) after PAA activation (Equation (7)) [31,49,50]. The decomposition of CH_3_COO^•^ can generate CO_2_ and CH_3_^•^, which could further react with oxygen to generate CH_3_OO^•^. The generated CH_3_^•^ and CH_3_OO^•^ showed low reactivity towards contaminants. On the other hand, Co(III) further reacted with PAA to generate CH_3_COOO^•^ (Equation (8)), another effective reactive organic radical contributed to the rapid oxidation of SMX; while Co(III) was reduced to Co(II), and further participated in the PAA activation. Overall, Co acted as a catalyst role in the PAA activation to generate CH_3_C(=O)O^•^ and CH_3_C(=O)OO^•^ for SMX oxidation.

(7) $$\equiv {\mathrm{Co}}^{2+}{+\mathrm{CH}}_{3}C(=O)\mathrm{OOH}\;\to \;\equiv {\mathrm{Co}}^{3+}{+\mathrm{CH}}_{3}{C(=O)O}^{•}+{\mathrm{OH}}^{-}$$

(8) $$\equiv {\mathrm{Co}}^{3+}{+\mathrm{CH}}_{3}C(=O)\mathrm{OOH}\;\Leftrightarrow \;\equiv {\mathrm{Co}}^{2+}{+\mathrm{CH}}_{3}{C(=O)\mathrm{OO}}^{•}{+H}^{+}$$

### 2.5. Effects of Water Matrices

**Figure 6:**
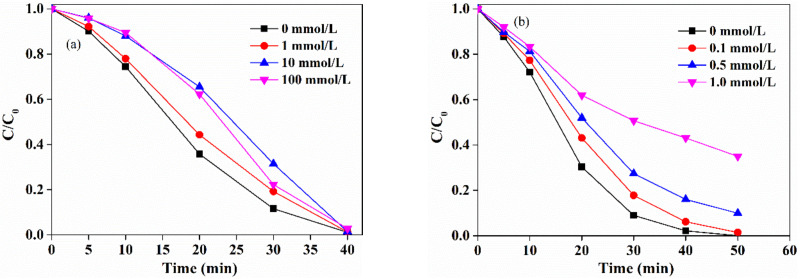

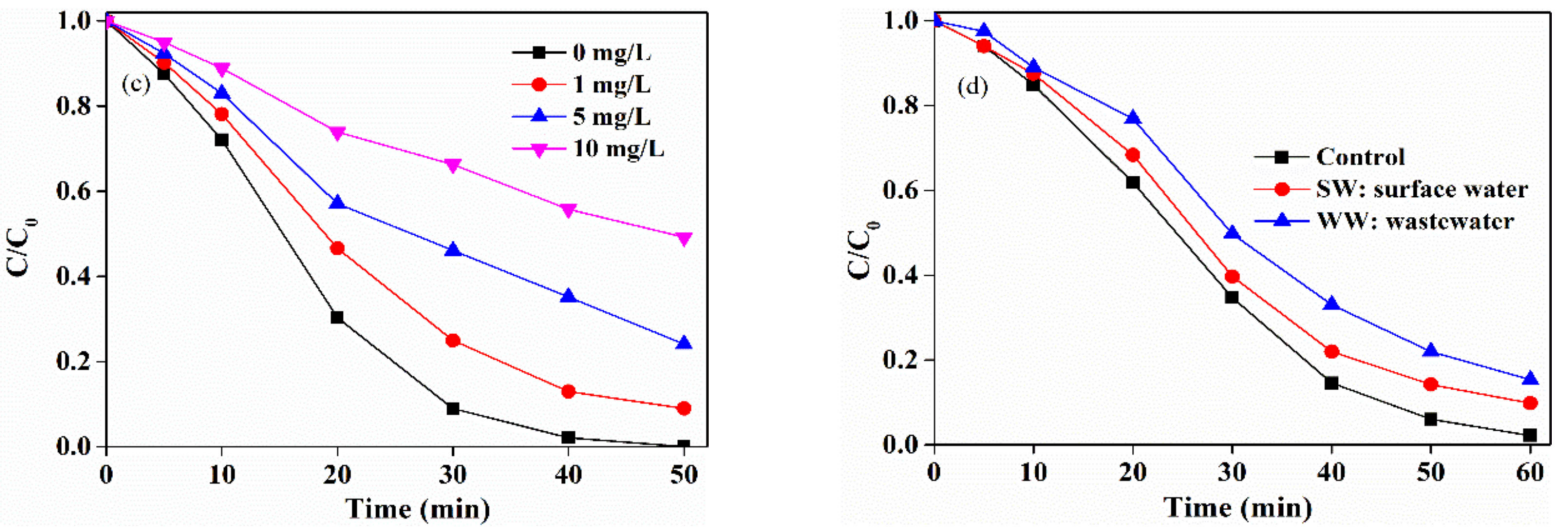
Effect of (**a**) Cl^-^, (**b**) HCO_3_^−^, (**c**) humic acid and (**d**) real water on the degradation of SMX by the LaCoO_3_/PAA system. LaCoO_3_ = 20 mg/L, SMX = 50 μM, PAA = 660 μM, pH 7.0, 25 °C.

The effects of water matrices, such as common anions and natural organic matter, were evaluated on SMX degradation in the LaCoO_3_/PAA system. As shown in Figure 6a, the presence of Cl^−^ (0–20 mM), a pervasive anion in natural water, slightly inhibited the degradation of SMX in the LaCoO_3_/PAA system. Cl^−^ is a common radical scavenger in AOPs and can react with the radical species to generate less reactive chlorine radicals, such as Cl^•^ and Cl_2_^•−^. The consumption of reactive radicals by Cl^−^ could reduce the reaction between R-O^•^ with SMX and thus reduce the degradation efficiency of SMX.

HCO_3_^−^/CO_3_^2−^ was reported as a common radical scavenger in PMS-based AOPs and the Co(II)/PAA system [51]; thus, the effects of HCO_3_^−^/CO_3_^2−^ on the degradation of SMX was also studied in the LaCoO_3_/PAA system. As shown in Figure 6b, HCO_3_^−^/CO_3_^2−^ could significantly inhibit the SMX oxidation in the LaCoO_3_/PAA system, and the inhibitory effect rapidly increased with an increasing concentration of HCO_3_^−^/CO_3_^2−^. Noting that the reaction was maintained at pH 7.0 with phosphate buffer, HCO_3_^−^ is the dominant species in the system due to the pK_a1_ = 6.4. Although HCO_3_^−^ can significantly quench ^•^OH, ^•^OH just played a minor role in the LaCoO_3_/PAA system for SMX degradation. Meanwhile, HCO_3_^−^ was reported to show very low reactivity towards R-O^•^, according to the phenomenon that the contaminant degradation in the UV/PAA + TBA system was not affected by HCO_3_^−^ [19]. Thus, the reactions between HCO_3_^−^ and the reactive radicals (^•^OH and R-O^•^) would not be the primary reason for the significant inhibition of SMX degradation in the LaCoO_3_/PAA system. The inhibitory effect could be attributed to the influence of HCO_3_^−^ on the interaction between PAA and LaCoO_3_ by competitive adsorption.

The effects of natural organic matter on SMX degradation were studied by adding a certain amount of humic acid (HA) in the LaCoO_3_/PAA system. As shown in Figure 6c, the presence of HA (0–10 mg/L) showed an apparent inhibition on SMX degradation in the LaCoO_3_/PAA system. To be specific, the degradation of SMX significantly decreased from 100% to 51% after 50 min as the concentration of HA increased from 0 to 10 mg/L. HA was previously reported to react with R-O^•^, with the rate constants reaching 10^4^ L mg^−1^ s^−1^. Hence, the addition of HA could compete with SMX towards R-O^•^ and thus reduce the degradation efficiency of SMX in the LaCoO_3_/PAA system.

The impact of real water matrices on the degradation of SMX was further explored in LaCoO_3_/PAA. The characteristics of the surface water (SW) and wastewater (WW) were listed in Table 1. As shown in Figure 6d, the degradation of SMX was inhibited in SW and WW, with the degradation efficiency moderately decreased to 90% and 85% after 60 min, respectively. Hence, some matrices in the real water sample might impose an adverse effect on SMX degradation in the LaCoO_3_/PAA system. Based on the above analysis, the slight inhibition that occurred in SW and WW matrices was likely to primarily contribute to the scavenging of R-O^•^ by organic matters.

### 2.6. Reusability of LaCoO_3_

**Figure 7:**
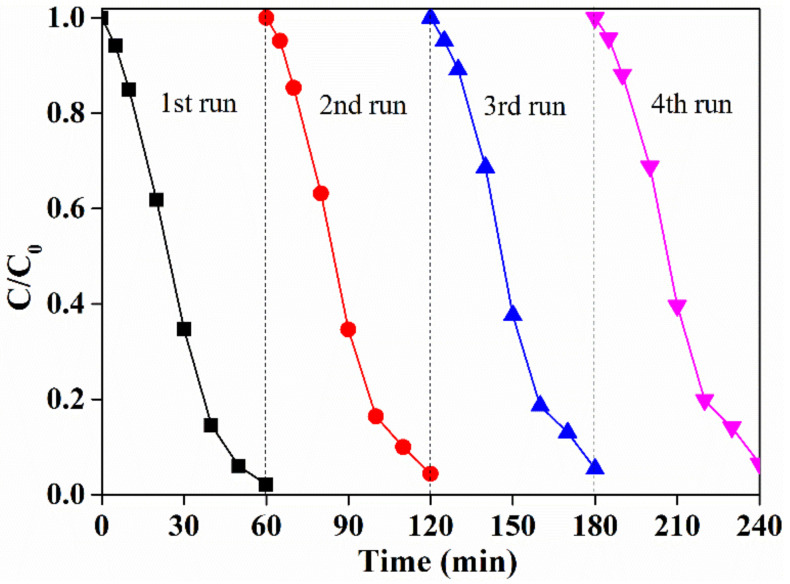
Reusability of LaCoO_3_. LaCoO_3_ = 20 mg/L, SMX = 50 μM, PAA = 660 μM, pH 7.0, 25 °C. black square: 1st run, red circle: 2nd run, blue up triangle: 3rd run, magenta down triangle: 4th run.

The reusability is crucial for the application of a catalyst in the heterogeneous reactions. LaCoO_3_ was reused to evaluate its performance on PAA activation after the reaction. As shown in Figure 7, an excellent performance of LaCoO_3_ in PAA activation to degrade SMX was still observed after four runs. Almost all SMX was completely removed in each run with a slight downward trend in the removal rates (from 98% in the 1st run to 95% in the 4th run). Moreover, the low leaching of Co under a neutral pH condition after the reaction by about 60 min also indicated the high structural stability of the prepared LaCoO_3_ during the reaction. Hence, the prepared LaCoO_3_ showed a high structural and chemical stability in PAA activation for the degradation of SMX.

## 3. Materials and Methods

### 3.1. Chemicals and Reagents

PAA solution (39% PAA and 6% H_2_O_2_ *w*/*w*), hydrogen peroxide solution (30% H_2_O_2_ *w*/*w*), sulfamethoxazole (SMX), cobaltous sulfate (CoSO_4_), cobaltous acetylacetonate (Co(C_5_H_7_O_2_)_2_), and cobaltic acetylacetonate (Co(C_5_H_7_O_2_)_3_) were purchased from Sigma-Aldrich (St. Louis, MO, USA). Oxone (KHSO_5_·0.5KHSO_4_·0.5K_2_SO_4_) and lanthanum nitrate hexahydrate (La(NO_3_)_3_·6H_2_O) were purchased from Aladdin Industrial Corporation. Cobalt nitrate hexahydrate (Co(NO_3_)_2_·6H_2_O), citric acid monohydrate (C_6_H_8_O_7_·H_2_O), sodium sulfate (Na_2_SO_4_), sodium nitrate (NaNO_3_), sodium chloride (NaCl), sodium bicarbonate (NaHCO_3_), ascorbic acid (AA), methanol (MeOH), and *tert*-butanol (TBA) were purchased from Sinopharm Chemical Reagent Co., Ltd., China. All solutions were prepared with ultrapure water from a Milli-Q academic (Millipore) system (MILLI-Q^®^ INTEGRAL 5, 18.2 MΩ cm).

### 3.2. Preparation and Characterization of LaCoO_3_

LaCoO_3_ was synthesized by a sol-gel process. Briefly, the mixtures of metal nitrates and citric acid were stirred for 12 h and then heated at 100 °C to remove water in the oil bath. The resultant sticky gel was subsequently oven-dried at 100 °C overnight. The obtained spongy material was then grinded into fine powders and calcined for 7 h in a muffle furnace to form the desired perovskite structure. After cooling, the sample was washed and dried overnight.

Transmission electron microscope (TEM) and selected-area electron diffraction (SAED) patterns were conducted on the field emission transmission electron microscope (Tecnai G2 F30, Eindhoven, Holland). The X-ray powder diffraction (XRD) analysis was characterized by Cu Kα irradiation (λ = 1.5406 Å) (Bruker D8-Advance, Karlsruhe, Germany). The surface area of LaCoO_3_ was analyzed by a Brunauer–Emmett–Teller (BET) analyzer (Micrometrics ASAP 2020, Norcross, GA, USA). The zeta potential analysis was acquired through a Malvern Zetasizer with a Zetasizer (Nano ZS90, Malvern, UK) at 25 °C.

### 3.3. Experimental Procedures

The batch experiments were conducted in 100 mL amber glass bottles. The solution was mixed by magnetic stirring at room temperature (25 °C). The reactions were initiated by adding PAA (660 μM) to the solution containing SMX (50 μM) and LaCoO_3_ (20 mg/L). Sample aliquots were taken at the predetermined time intervals and subsequently quenched with excess Na_2_S_2_O_3_ immediately. Afterwards, the quenched sample was filtered through a 0.22 μm membrane and analyzed within 24 h. The initial pH of the solution was adjusted by H_2_SO_4_ and NaOH to the designated value. Control experiments without PAA or LaCoO_3_ were conducted to evaluate the contributions of PAA or LaCoO_3_ alone for the degradation of SMX. Radical scavenging experiments were conducted with addition of 500 mM of alcohols (tert-butyl alcohol (TBA) or methanol (MeOH)). In order to assess the reusability of LaCoO_3_ in the activation of PAA, the catalyst was collected by centrifugation and then washed with methanol and ultrapure water. Afterwards, the solid was dried at 60 °C overnight and four runs were carried out to evaluate the reusability. All the experiments were conducted in duplicate or more.

### 3.4. Analytical Methods

SMX was analyzed by high-performance liquid chromatography (HPLC 1290, Agilent Technology, Santa Clara, CA, USA) equipped with a Zorbax SB-C18 column (4.6 × 250 mm, 5 μm) and a UV detector at a wavelength of 265 nm. The flow rate was set at 1 mL min^−1^ and the injection was set at 20 μL. The mobile phase was the mixture of acetonitrile and water containing 0.4% acetic acid (70:30).

The PAA concentration in the solution was quantified by the N,N-diethyl-*p*-phenylenediamine (DPD) colorimetric method [19]. Briefly, the PAA solution was sampled and then was immediately mixed with excess KI (60 mM), DPD (2.8 mM), and phosphate buffer (0.5 M at pH 6.5) and measured at 515 nm on a UV-visible spectrophotometer (Beckman DU 520, Beckman Coulter, Inc., Fullerton, CA, USA).

## 4. Conclusions

LaCoO_3_ was successfully synthesized and used for PAA activation to degrade SMX. The rapid degradation of SMX was observed with the heterogeneous activation of PAA by LaCoO_3_ under a neutral pH condition. Cl^−^ was slightly inhibited by the degradation of SMX, whereas HCO_3_^−^ and HA significantly reduce SMX in the LaCoO_3_/PAA system. LaCoO_3_ showed high structural stability and reusability after consecutive runs with low Co leaching under a neutral condition. Organic radicals, e.g., CH_3_C(=O)O^•^ and CH_3_C(=O)OO^•^ were proposed as the primary radical species responsible for SMX degradation in the LaCoO_3_/PAA system. This work provides an emerging AOP relying on organic radicals to degrade organic contaminants in the wastewater treatment.

## Figures and Tables

**Table 1:** Characteristics of water samples.

Sample	TN (mg/L)	TP (mg/L)	pH	UV254	TOC (mg/L)	Cl^−^ (mg/L)
SW	0.81	0.07	7.8	0.13	3.1	32.6
WW	11.1	0.84	7.1	0.41	10.6	92.3

Note: SW: surface water; WW: wastewater.
